# Two-Dimensional Analysis of the Size of Nasopharynx and Adenoids in Non-Syndromic Unilateral Cleft Lip and Palate Patients Using Lateral Cephalograms

**Published:** 2018-05

**Authors:** Sarvin Sarmadi, Javad Chalipa, Behrad Tanbakuchi, Maryam Javaheri Mahd, Maryam Nasiri, Mohammad Reza Mehtari

**Affiliations:** 1 Assistant Professor, Department of Orthodontics, School of Dentistry, Tehran University of Medical Sciences, Tehran, Iran; 2 Postgraduate Student, Department of Orthodontics, School of Dentistry, Tehran University of Medical Sciences, Tehran, Iran; 3 Dentist, Private Practice, Tehran, Iran

**Keywords:** Cleft Lip, Cleft Palate, Nasopharynx, Adenoids, Cephalometry

## Abstract

**Objectives:**

Cleft lip and palate (CLP) is the most common congenital anomaly of the head and neck region. The upper airway in CLP patients is affected by retarded maxillary growth. Small size of the nasopharynx can also lead to mouth breathing. This study aimed to compare the size of nasopharynx and adenoids in non-syndromic unilateral CLP (NSUCLP) patients and healthy controls two-dimensionally on lateral cephalograms.

**Materials and Methods:**

This retrospective study was performed on 30 children with NSUCLP (mean age of 11.3 years) and 30 sex- and age-matched healthy controls with class I skeletal relationship. The bony boundaries of the nasopharynx, nasopharyngeal airway and adenoids were outlined on lateral cephalograms and their surface area was calculated and compared between the two groups. The percentage of nasopharynx occupied by the adenoids was calculated for each individual and compared between the two groups using independent t-test.

**Results:**

Size of nasopharynx in NSUCLP children was significantly smaller than that in healthy controls (P=0.0001). Size of adenoids was significantly larger in NSUCLP children (P=0.0001). Size of nasopharyngeal airway was smaller in NSUCLP patients than controls (P=0.0001). Percentage of nasopharynx occupied by the adenoids was significantly greater in NSUCLP patients (P=0.0001).

**Conclusions:**

The size of nasopharynx is smaller while the size of adenoids is larger in NSUCLP children compared to healthy controls; this can lead to mouth breathing and velopharyngeal incompetence.

## INTRODUCTION

Oral clefts are the most common craniofacial deformities accounting for 15% of all congenital anomalies [1]. In the United States, 7.75 out of every 10,000 babies born have cleft lip and palate (CLP). This rate is 7.94 per 20,000 live births in other parts of the world [2]. The etiology of CLP is multifactorial and includes both genetics and environmental factors such as drug use, tobacco consumption and radiation [3]. Surgical treatment of CLP is often started in early infancy and continues to adulthood [4]. Upper airway status is important for orthodontists that study the relationship of facial type and airway morphology [5,6]. Growth and development of nasopharyngeal airway follows a fast pace until 13 years of age [7,8] and then slows down [9]. Taylor et al. [8] reported that the anterior-posterior dimension of the upper airway increases between the ages of 6 to 9 years in the first phase and then continues to grow between the ages of 12 to 15 years. Slight changes occur between the ages of 9 to 12 years [8]. Adenoids are comprised of lymphoepithelial tissue and are part of the Waldeyer’s tonsillar ring in the pharynx; they play an important role in the immune system [10]. Linder-Aronson [10] studied the details of adenoid growth in normal individuals on lateral cephalograms and reported that they often grow during childhood. However, their pathological changes are common between the ages of 2–12 years. Their size decreases during the adolescence and puberty, which is often concomitant with an increase in size of nasopharynx. Thus, adenoids occupy a smaller percentage of the nasopharyngeal space [9,11].

Hypertrophy of adenoids may cause complete or partial obstruction of the nasopharyngeal space, which may compromise nasal breathing. Discrepancy between the growth of adenoids and nasopharyngeal airway may be related to the different growth patterns of the bones in the nasopharynx and attached tonsillar tissues. Obstruction of the nasopharynx eventually leads to chronic mouth breathing. This condition is referred to as respiratory obstruction syndrome [9]. Previous studies highlighted significant differences in facial growth patterns, especially in the maxilla, between CLP patients and normal subjects [5,12–14]. It has been reported that CLP patients have a smaller nasal airway than healthy controls, which makes them prone to mouth breathing [15–17]. CLP patients and normal individuals have differences in upper airway and the surrounding tissues in terms of both morphology and function [18]. Osborne et al. [19] discussed that anomalies increasing the pharyngeal depth can enhance the occurrence of velopharyngeal incompetency. Significant effect of adenoids in CLP children on the correct function of velopharynx has been previously documented [20,21]. Presence of adenoid tissue allows velopharynx closure to occur more anteriorly in the nasopharyngeal space [18]. Impaired function of velopharynx is a main problem in CLP patients. In most cases, complete velopharynx closure occurs after the primary repair of the palate; however, nasal speech remains in some cases until years later [22]. Rose et al, [23] also reported higher frequency of mouth breathing, snoring and hypopnea during sleep in CLP patients compared to healthy controls. Assessment of the morphology of the pharyngeal airway is also important in CLP patients. Most previous studies marked lines on lateral cephalograms and assessed changes in morphology by linear measurements in one or two dimensions [16,17, 24]. Lateral cephalograms are suitable for assessment of the bony boundaries of the nasopharynx, nasopharyngeal airway, nasopharyngeal soft tissue, pharyngeal wall and adenoid tissue. Although lateral cephalometry has limitations such as superimposition of structures and two-dimensional view of three-dimensional structures, it is suitable for such assessments since it is easily accessible and low-cost and has low patient radiation dose and adequately high accuracy [25]. There is gap of information about the size of adenoids in the Iranian preadolescent CLP patients compared to normal individuals, and the relationship of adenoids and nasopharynx has not been well evaluated. Search of the literature yielded no study in this respect conducted in Iran and the existing studies on other populations have reported controversial results. Considering all the above, this study aimed to compare the size of nasopharynx and adenoids in non-syndromic unilateral CLP (NSUCLP) patients and healthy controls two-dimensionally on lateral cephalograms.

## MATERIALS AND METHODS

This retrospective case-control study was conducted on 60 medical records retrieved from the archives of the Orthodontics Departments of Schools of Dentistry of Shahid Behshti and Tehran universities. Sample size was calculated to be 30 in each of the two groups according to a previous study [22] assuming alpha=0.01 and beta=0.0. A total of 60 medical records of children between 9–12 years were evaluated. The test group included 12 females and 18 males in the age range of 9 to 12 years (mean age of 11.3 years) with NSUCLP who had digital lateral cephalograms in their medical files. All patients had undergone surgical repair of the CLP. Most of them had undergone surgical repair of the lip between 3–6 months and surgical repair of the palate between 9–12 months. None of the patients had undergone adenoidectomy, tonsillectomy or pharyngoplasty according to their medical records. All radiographs had been obtained before the initiation of orthodontic treatment.

Thirty age- and sex-matched healthy individuals (12 females and 18 males, mean age of 11.7 years) who had lateral cephalograms were considered as controls. Cephalograms of the control group had been taken before their orthodontic treatment. They had normal craniofacial morphology, class I skeletal relationship (ANB=2.51±0.85°, Wit’s appraisal= −0.43±0.99 mm), no jaw deformity and no mouth breathing. All medical records of CLP patients and normal individuals had been collected by an orthodontist. Cephalograms had been taken in natural head position. All lateral cephalograms were traced on a negatoscope using a pencil (0.5 mm) and acetate cellulose tracing paper by two researchers, and the bony boundaries of the nasopharynx, soft tissue contour of the nasopharynx, contour of the adenoids and soft tissue contour were outlined. Due to optimal contrast, the boundaries between tissues were clearly visible and traceable. For the purpose of calibration, tracing of 10 randomly selected lateral cephalograms was double checked by an oral and maxillofacial radiologist. During tracing, the magnification scale of each image was also recorded on the tracing paper to prevent magnification error.

**Fig. 1:**
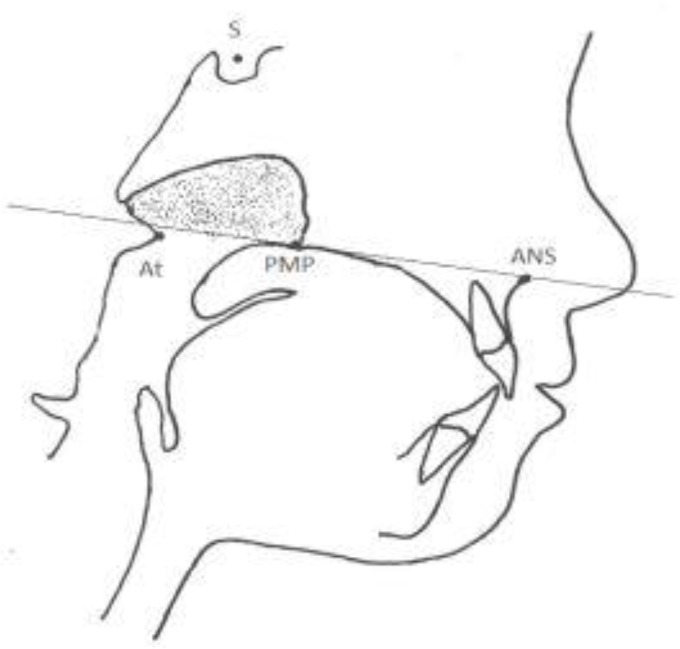
Bony boundaries of the nasopharynx

**Fig. 2:**
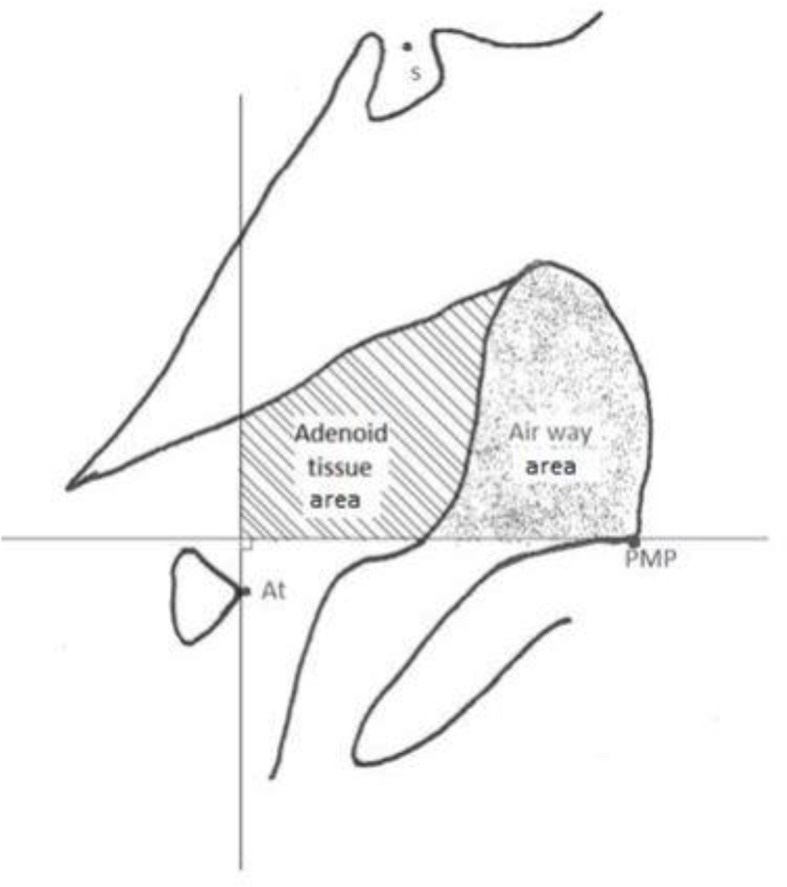
Boundaries of the adenoid tissue

**Fig. 3:**
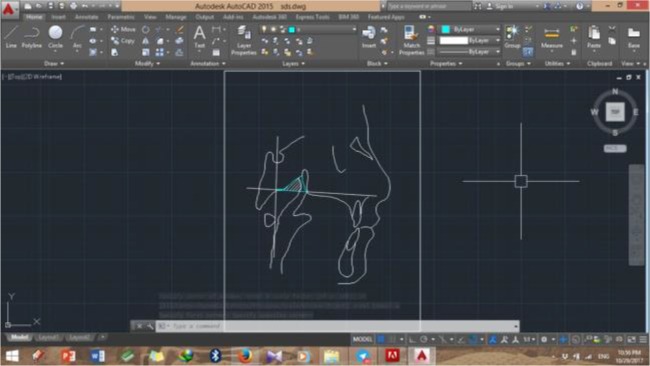
Bony nasopharynx of the test group in AutoCAD software

**Fig. 4:**
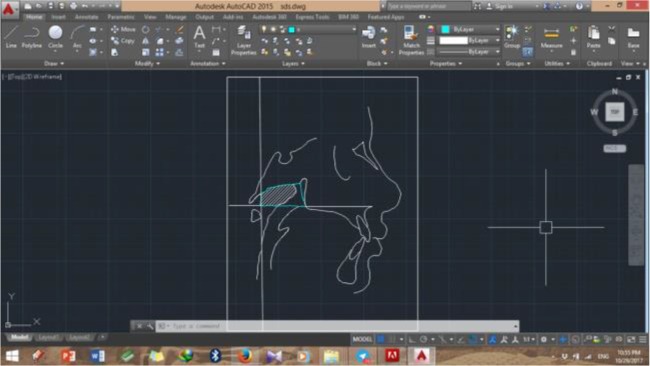
Bony nasopharynx of the control group in AutoCAD software

**Fig. 5:**
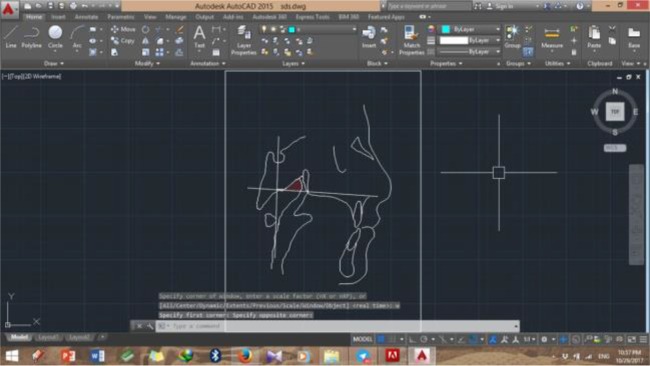
Adenoid tissue of the test group in AutoCAD software

**Fig. 6:**
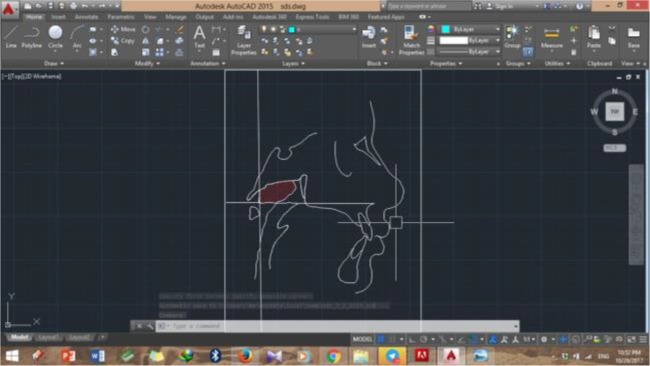
Adenoid tissue of the control group in AutoCAD software

A shown in Figure 1, the bony boundaries of the nasopharynx were outlined. The palatal plane was drawn by connecting the anterior nasal spine and the pterygomaxillary point, and the space above the palatal plane was considered as the bony boundaries of the nasopharynx [22]. Soft tissue of the posterior pharyngeal wall was also traced. The adenoid tissue was also traced as shown in Figure 2 (space confined to the anterior contour of the adenoid tissue). For this purpose, a line was drawn from point (At), which is the most anterior point of the anterior tubercle of the atlas vertebra, perpendicular to the palatal plane [18]. The traced cephalograms were scanned by a scanner (N9120 ScanJet; HP, CA, US) and transferred to AutoCAD version 2013 software (Autodesk, CA, USA) (Figs. 3–6). The same magnification scale was considered for all cephalograms. The surface area of the nasopharynx and adenoid tissue was separately calculated [9].

Also, the percentage of the asopharyngeal space occupied by the adenoid tissue was calculated and the remaining airway space was calculated for all patients. Data were analyzed using SPSS version 22 (SPSS Inc., IL, USA). Normal distribution of data was assessed using the Kolmogorov-Smirnov test. The bony boundaries of the nasopharynx, adenoid tissue, nasopharyngeal airway and percentage of nasopharyngeal space occupied by the adenoid tissue were compared between the NSUCLP patients and healthy controls using independent t-test.

## RESULTS

The Kolmogorov-Smirnov test showed that all variables had a normal distribution in both groups (P>0.05). Table 1 shows the mean surface area of the nasopharynx, adenoid tissue, nasopharyngeal airway and percentage of nasopharynx occupied by the adenoid tissue in the two groups. As shown in Table 1, the mean size of the nasopharynx was significantly greater in the healthy control group (P<0.0001). The mean size of the adenoid tissue was significantly greater in the NSUCLP patients compared to the healthy controls (P<0.0001). The mean size of the pharyngeal airway space was significantly greater in the healthy control group (P<0.0001). The mean percentage of nasopharynx occupied by the adenoid tissue was significantly greater in NSUCLP patients than in healthy controls (P<0.0001).

**Table 1.** Summary of the results

**Parameter**	**Mean± Standard deviation**		**P-value**

	**UCLP (n=30)**	**Control (n=30)**	
Surface area of nasopharynx (mm^2^)	483.929±36.160	528.473±27.810	<0.001
Surface area of adenoid tissue (mm^2^)	297.616±22.659	253.001±19.901	<0.001
Surface area of nasopharyngeal airway (mm^2^)	186.313±44.737	275.473±35.141	<0.001
Percentage of nasopharynx occupied by adenoid tissue (%)	61.880±7.020	48.020±6.360	<0.001

## DISCUSSION

The adenoids also known as the pharyngeal tonsils affect the shape and size of the nasopharynx and play a role in velopharyngeal competency before puberty. Normally, adenoids are large in children and gradually shrink by an increase in age. Controversy exists regarding the etiologic role of hypertrophic adenoids in childhood and their effect on facial growth and dentoskeletal anomalies [26]. Hypertrophic adenoids at any age can interfere with the nasal air flow and cause hyponasality [27]. Adenoid tissue grows fast and may occupy half of the nasopharyngeal space by 2–3 years of age. In patients with cleft palate, adenoids are often larger than those in normal individuals, which is often compensatory due to decreased depth of larynx in order to allow velopharyngeal competence [28]. Imamura et al. [18] reported that children with NSUCLP have larger adenoids and smaller upper airways than their healthy peers; our study on the 9–12 year-old Iranian patients showed the same results.

Adenoids undergo atrophy by an increase in age. In normal individuals, the posterior wall of the pharynx approximates the soft palate, which is a physiological compensatory act for closure of the velopharynx. This physiological compensation does not occur in CLP patients [27]. Mason and Warren [29] assessed the nasality of voice and airflow and took cephalometric radiographs and concluded that after natural atrophy of the adenoids, about one-third of patients develop hypernasality. As mentioned earlier, patients with repaired CLP and normal speech are prone to velopharyngeal insufficiency after removal of adenoids [27]. Celikoglu et al. [30] assessed the nasopharyngeal and oropharyngeal sizes and total airway volume of CLP patients using cone beam computed tomography (CBCT) and concluded that these sizes were smaller in CLP patients than in normal individuals. Similarly, the current study determined the size of nasopharynx and nasopharyngeal airway in NSUCLP patients and healthy controls and showed that the size of nasopharynx and nasopharyngeal airway in NSUCLP patients was significantly smaller than that in healthy controls. Also, the percentage of nasopharynx occupied by the adenoid tissue was significantly greater in NSUCLP patients.

Wada et al, [22] in their longitudinal study measured the linear size of the nasopharynx and form of the cranial base and cervical vertebrae in NSUCLP patients and compared these parameters with those of normal individuals. They found no significant difference in the form of cranial base and cervical vertebrae between the two groups. But the posterior maxillary growth was significantly retarded in CLP patients, irrespective of age, in both vertical and horizontal dimensions compared to healthy age-matched controls. They did not measure the size of the adenoid tissue and did not evaluate its relation to nasopharynx. Although the definitions of nasopharynx and adenoids in their study were in line with those in our study, they only calculated the linear distance between the reference points and outlined the nasopharynx as a triangle with (HO), pterygomaxillary point and (At) as its three corners. In the current study, the nasopharynx was precisely outlined according to the soft tissue boundaries. However, the results of Wada et al. [22] were different from ours. But, in both studies, the size of nasopharynx in CLP patients was smaller than that in healthy controls.

Our study showed that the adenoid tissue in children with UCLP occupied a higher percentage of nasopharynx. This decreases the size of airway and complicates nasal breathing. As the result, the children gradually start mouth breathing. Oosterkamp et al. [13] reported that adults with CLP and obstructive sleep apnea have similar craniofacial and pharyngeal airway morphology except that the growth of the maxilla is significantly retarded in CLP patients. In patients with obstructive sleep apnea, the craniocervical angle increases and the hyoid bone is positioned more posteriorly [13]. High prevalence of obstructive sleep apnea in CLP children is due to the impairment of muscles supporting the palate as well as some other structural anomalies of the maxilla and mandible [14]. MacLean et al. [31] evaluated 55 patients with CLP and 113 healthy controls in terms of mouth breathing during sleep and concluded that the only difference between the two groups was enlarged tonsils and higher prevalence of snoring and sleep apnea in CLP children. Mouth breathing can cause frequent upper airway and otolaryngological infections [16,32].

After reparative surgery of the palate, difference between the growth of the flap and the surrounding nasopharyngeal tissue is a possible reason for nasopharyngeal incompetency, because the flap has a retarded growth due to surgical scar, which leads to velopharyngeal incompetency [33]. The effects of surgical techniques and timing of surgical procedures on the growth of nasopharynx have not been well determined. However, differences have been reported in nasopharyngeal growth in CLP patients and healthy individuals [20,22]. Some studies have suggested closure of the cleft as soon as possible due to the significance of speech while some others prefer delaying the surgery due to the significance of maxillary growth [18]. Small sample size was a limitation of the current study. Only 30 patients with NSUCLP were enrolled during two years. Use of CBCT could have provided more valuable information. However, aside from the high cost of CBCT, a full-skull CBCT has high patient radiation dose and is not commonly requested for NSUCLP orthodontic patients. Moreover, finding healthy controls with available CBCT scans was very difficult. Thus, lateral cephalograms available in patient files were used in the current study. However, previous studies used trigonometric relationships to calculate the size of the nasopharynx and adenoids; whereas, we precisely outlined these areas on lateral cephalograms and measured the surface areas using AutoCAD 2013 software.

To sum up, in CLP patients, adenoidectomy and maxillary expansion can enhance nasal breathing; however, some other considerations should also be taken into account prior to surgical treatment planning.

## COUCLUSION

Size of the nasopharynx is smaller and size of the adenoid tissue is larger in NSUCLP children compared to normal controls; this can lead to mouth breathing and velopharyngeal incompetency.
